# Analysis of heavy metal, rare, precious, and metallic element content in bottom ash from municipal solid waste incineration in Tehran based on particle size

**DOI:** 10.1038/s41598-023-43139-1

**Published:** 2023-09-25

**Authors:** Masoumeh Beikmohammadi, Kamyar Yaghmaeian, Ramin Nabizadeh, Amir Hossein Mahvi

**Affiliations:** Department of Environmental Health Engineering, School of Public Health, Tehran University of Medical Sciences, Tehran, Iran

**Keywords:** Environmental sciences, Environmental social sciences, Solid Earth sciences

## Abstract

Waste incineration is increasingly used worldwide for better municipal solid waste management and energy recovery. However, residues resulting from waste incineration, such as Bottom Ash (BA) and Fly Ash (FA), can pose environmental and human health risks due to their physicochemical properties if not managed appropriately. On the other hand, with proper utilization, these residues can be turned into valuable Municipal metal mines. In this study, BA was granulated in various size ranges (< 0.075 mm, 0.075–0.125 mm, 0.125–0.5 mm, 0.5–1 mm, 1–2 mm, 2–4 mm, 4–16 mm, and > 16 mm). The physicochemical properties, heavy metal elements, environmental hazards, and other rare and precious metal elements in each Granulated Bottom Ash (GBA) group from Tehran's waste incineration were examined using ICP-MASS. Additionally, each GBA group's mineralogical properties and elemental composition were determined using X-ray fluorescence (XRF) and X-ray diffraction (XRD). The results showed that the average concentration of heavy metals in GBA, including Zn (1974 mg/kg), Cu, and Ba (790 mg/kg), Pb (145 mg/kg), Cr (106 mg/kg), Ni (25 mg/kg), Sn (24 mg/kg), V (25 mg/kg), As (11 mg/kg), and Sb (29 mg/kg), was higher in particles smaller than 4 mm. Precious metals such as gold (average 0.3 mg/kg) and silver (average 11 mg/kg) were significantly higher in GBA particles smaller than 0.5 mm, making their extraction economically feasible. Moreover, rare metals such as Ce, Nd, La, and Y were detected in GBA, with average concentrations of 24, 8, 11, and 7 mg/kg, respectively. The results of this study indicated that BA contains environmentally concerning metals, as well as rare and precious metals, with high concentrations, especially in particles smaller than 4 mm. This highlights the need for proper pre-treatment before using these materials in civil and municipal applications or even landfilling.

## Introduction

The production of municipal solid waste (MSW) is rapidly increasing worldwide due to increased population, rapid urbanization, and improved living standards^1,2^. According to a World Bank report and conducted studies, global MSW production was 2.24 billion tons in 2020, which is expected to increase to 3.88 billion tons by 2050^2–4^. MSW mainly consists of food waste, paper, plastic, wood, and textiles, with quantities varying depending on people's lifestyles and cultures, waste management policies, and regulations in each society^5,6^. MSW treatment processes have improved over time, and today, environmental concerns arising from MSW disposal can be easily overcome through thermal processes, anaerobic digestion, and waste landfill bioreactors while generating energy^1^.

Incineration and landfilling are the primary approaches for treating and disposing of MSW worldwide^7^. Landfilling methods have lost their appeal due to disadvantages such as the need for considerable land area, the loss of primary use after landfill closure, and potential soil and groundwater contamination with heavy metals^8^. However, incineration is a thermal technology that offers benefits such as volume, mass, and organic matter reduction of MSW by 85–90%, 60–90%, and 100%, respectively, harmlessness, high treatment efficiency, and minimal land area required for waste incineration facilities compared to landfilling^9,10^. As a result of incineration, MSW can be considered a renewable energy source due to its high potential for energy production^10–13^. Therefore, most developed countries have chosen incineration as an effective method for treating and disposing of MSW^14^. However, incineration is not the final stage of MSW disposal. Although organic materials are completely oxidized to carbon dioxide and water vapor and eliminated during incineration, significant amounts of inorganic compounds remain in the residues resulting from incineration, causing environmental concerns due to their release into the air or burial in the ground. These residues are typically disposed of in landfills, potentially leading to soil and water pollution^15^.

During waste incineration, two main products are produced: Bottom Ash (BA), resulting from burning waste in the combustion chamber, and Fly Ash (FA), which is released through the flue gas^16,17^. Their volume accounts for approximately 25% of the input waste volume in the incinerator, which is usually disposed of in landfills^18^. BA constitutes about 80–95% of the total ash weight, has a wide range of particle sizes, from silt/clay to gravel, and can be used as secondary raw material^2,17,19^. It contains various compounds such as melted glass, ceramics, unburnt organic matter, silicates, and metallic and non-metallic compounds^20,21^. This heterogeneous composition forms during waste combustion at temperatures between 850 and 1000 °C in the incinerator^21^. The composition of BA varies from country to country due to geographical conditions, waste production area, season, vegetation characteristics, and lifestyle^22,23^. Additionally, several factors affect the BA compound, including furnace configuration, combustion temperature, quenching process, and incineration waste composition^24,25^. Recently, extensive research has been conducted to increase the utilization rate of Bottom Ash (BA) in civil and municipal activities such as cement production, road construction, and land reclamation to reduce the consumption of natural resources. The results showed that the engineering properties of soil amended with BA have significantly improved^17,18,26^. Studies also indicate that particle size distribution is one of the main parameters in selecting road construction materials. BA particle sizes cover a wide range, influenced by factors such as time, location, and waste origin, with particles around 9.5 mm suitable for road construction^26^.

The granulation of BA particles increases the efficiency of metal recycling, and particles with smaller sizes, which are more likely to contain harmful environmental elements, are used less in construction and civil purposes^27^. Also, according to the size of the particles, the principal elements and metals in them are different. Al is present in particles of 6–20 mm (60%)^28^, copper mainly in particles less than 7 mm (70%)^29^, and a significant concentration of precious metals gold and silver in particles > 2 mm^30–32^. Also, with decreasing particle size, the amount of Cr, Pb, Ni, and Sb increased, and in 1–4 mm particles, due to mineralogical changes, the amount of copper and zinc increased significantly^33^. Particles smaller than 4 mm, which account for half of the weight of BA, contain valuable metals as well as harmful elements, which should be taken into account to be used in construction purposes^27^. Moreover, there are significant environmental concerns due to heavy and toxic metals, including zinc, chromium, nickel, cadmium, lead, copper, mercury, and tin, some of which are found in relatively high concentrations and their potential leakage into the environment^15,34^. Studies have shown that any change in environmental conditions can cause heavy metal leakage from BA into the soil, surface water, and groundwater, with potential human and environmental toxicity effects^26,35^. In his study, Abramov showed that the particles < 2 mm, which accounts for 46% of the ash, are the most likely to leak Pb, Zn, Cu, Ba, and Ni^15^. Nevertheless, considering recent advancements in ash treatment and recycling, a considerable portion of BA has the potential to be treated and reused, especially particles smaller than 4 mm, which primarily contain heavy metals^36^ and organic salts such as sulfates and chlorides^37^. For this reason, the amount of heavy metals in this part of the incinerator waste and their leakage rate are of particular importance and should be carefully investigated and evaluated^17,18,26^. Consequently, regulations have been established in some countries to determine the total concentration of heavy metals before use, and pre-treatment is necessary to reduce the leakage of heavy metals from BA before using it in municipal construction. In fact, before using BA in the environment, it must comply with the environmental regulations set by different countries^16^. Many studies have shown that the disposal of untreated BA in landfills can release dangerous environmental pollutants and high concentrations of heavy metals and salts into the environment^19,25^. Proper treatment of BA before use can reduce the release of these pollutants into the environment^38,39^. To this end, BA's physical and chemical properties need to be investigated. However, generalizing the physicochemical properties of BA from one location to another is not easily possible, as these properties depend on various factors such as operating conditions, input waste composition, type of incinerator, air pollution control system, and system design^1,11^.

It is considered that in Iran, especially in Tehran, the primary method of managing municipal solid waste is sanitary landfilling, and apart from recycling, other management methods have yet to develop much. In recent years, due to the limited land for disposal, using incineration methods has attracted managers' attention, especially in areas with high groundwater levels. In this method, in addition to reducing the volume and mass of input waste, it can also be used as an energy source for electricity generation. Municipal incineration technology has been launched in Tehran and is developing in our country. However, the problem is the management of bottom ash. Since no study has been conducted on the quality of ash produced by incinerators in Iran and Tehran, it was deemed necessary to thoroughly investigate the physicochemical properties of the residues from Tehran's municipal incineration to use them appropriately in the future.

Therefore, this study aimed to granulate the particles of waste incineration bottom ash in various size ranges < 0.075 mm, 0.075–0.125 mm, 0.125–0.5 mm, 0.5–1 mm, 1–2 mm, 2–4 mm, 16–4 mm and > 16 mm and determining the physicochemical properties, heavy metal elements, environmental hazards, other rare and precious metal elements and loss on ignition in each group of Granulated Bottom Ash (GBA) from Tehran's waste incineration and investigating the mineralogical properties of ash in order to better and optimally use the produced ash and compare results of this study with other studies. Physicochemical properties were determined by measuring heavy and precious metals' content using ICP-OES and ICP-MASS, and the mineralogical properties by X-ray fluorescence (XRF) analysis. X-ray diffraction (XRD) was used to determine the chemical species, total chlorine, and heavy metal sources in incinerator ash and the reduction resulting from combustion.

## Materials and methods

### Bottom ash sampling

The bottom ash sample was collected from the waste processing complex of Aradkouh in Tehran, the capital of Iran, during the spring of 2021 (1400). This waste incinerator accepts non-recyclable waste that has undergone recycling and processing procedures. The input waste mainly consists of food waste, plastics, rubber, leather, textiles, and a small amount of wood and other mixed municipal solid waste, which enters the incinerator at a minimum temperature of 900 °C and forms BA in the combustion chamber, which is periodically removed. 150 kg of samples were taken from various depths of the BA piles (1–2 m) stored outdoors, and the BA sample from this facility was collected.

The coarse metallic and non-metallic components were manually separated. The sample was then divided into five equal parts, and a sample was taken from each part, yielding 50 kg of sample in five airtight plastic buckets that were transported to the laboratory and stored at room temperature. Before determining the properties, 2 kg of BA was taken from each bucket, transferred to an empty container, and mixed. This mixture was placed under a hood for three days^15^ and then used for physicochemical analyses. Supplementary Table S1 online shows Tehran's waste incinerator's specifications and operating conditions.

### Physical properties of municipal solid waste incinerator bottom ash

#### Particle size distribution of bottom ash

Determining the particle size distribution and range of bottom ash (BA) particles is crucial for managing the ash effectively. A sieving technique can achieve this. The bottom ash sample collected from the waste incinerator was highly heterogeneous. Therefore, coarse metal and non-metal pieces were removed from the sample before analysis, and agglomerated and adhered pieces were broken apart. The sample was then placed in an oven at 105 °C until a constant weight difference was achieved. Subsequently, the sample was sieved using analytical stainless-steel vibratory sieves with sieve numbers 518, 4, 10, 18, 35, 120, and 200 at room temperature (23 ± 0.5 °C) according to ASTM C136-AASHTO T27 standards in size ranges of groups A: > 0.075, B: 0.075–0.125, C: 0.125–0.5, D: 0.5–1, E: 1–2, F: 2–4, G: 4–16, and H: < 16 mm^40^. The sieved samples were then placed in polypropylene containers and stored in a desiccator at room temperature for determining other properties and conducting other tests.

#### Moisture content of sieved bottom ash

A specific amount of Bottom Ash (BA) was weighed using a METTLER/AE200 laboratory balance and placed in an oven at 105 °C for 24 h to determine the moisture content.

After the specified time had elapsed, the crucibles were removed from the oven and placed in a desiccator until they cooled down. Then, they were re-weighed, and their moisture content was determined using formula (1).

1 $$ \% {\text{W}} = \, \left( {\left( {{\text{W}}_{{1}} - {\text{W}}_{{2}} } \right)/{\text{W}}_{{1}} } \right) \, *{1}00) $$

W = Moisture percentage; W_1_ = Weight of ash sample before drying; W_2_ = Weight of ash sample after drying.

#### Granulated bottom Ash pH

To determine the pH of Granulated Bottom Ash (GBA), suspensions of air-dried GBA and deionized water produced by the Human Power III/Scholar-UV, VER 1.0 model device made in Korea were prepared at a 1:2.5 ratio (10 g of ash were added to every 25 mL of deionized water)^15^. These suspensions were poured into glass bottles with lids, sealed to prevent water evaporation and CO_2_ entry, and placed on an orbital shaker at 150 rpm at room temperature (23 ± 0.5 °C)^41^. The pH levels were measured at 1 h (pH/1 h), 24 h (pH/24 h), and 7 days (pH/7 day) with three repetitions, using a Kent EIL 7020 tabletop pH meter.

### Chemical properties of granulated bottom ash

#### Metallic and heavy metal content of granulated bottom ash

The metallic elements, heavy metals, and rare and precious metals present in each group of GBA were extracted. Their concentrations were determined using the Inductively Coupled Plasma Spectroscopy (ICP-OES device Spectro Arcos model, Company: SPECTRO, made in Germany, for determining major elements And ICP-MASS- device, Agilent 7500 model, made in the USA in 2001 for determining trace elements), according to standard metal methods^27,30,42^. These elements were investigated due to environmental concerns and potential damages^41^.

#### Elemental analysis of size-granulated bottom ash content

X-Ray Fluorescence (XRF) spectroscopy was conducted using an XRF instrument (model 840ERL) manufactured in the United States to determine the crystal phase properties and chemical composition of the main elements in the GBA samples. The particle size of the bottom ash was first reduced to less than 2 mm using a ball mill to perform this analysis.

#### Loss on ignition analysis of size-granulated bottom ash content

The samples were heated at 950 °C for 1.5 h to determine the Loss On Ignition of bottom ash particles. The analysis was subsequently carried out based on the ASTM E 1621–13 standard^43^.

#### Mineralogy of size-granulated bottom ash

The mineral properties and types of oxides present in the ash structure can influence the purification method. The mineralogy and types of oxides in the ash were studied using X-Ray Diffraction (XRD) technique^44^. This study used an XRD device (model X'PERT Pro MPD) manufactured by PANalytical in the Netherlands in 2009 to determine the mineral compositions and oxides in the BA samples. The mineralogy properties of the ash were determined at an angle of 2θ = 2-100º, using a Cu anode with a voltage of 40 kV and a current intensity of 30 mA, according to the BS EN 13925-1 standard^45^.

## Results and discussion

### Physical properties of granulated bottom ash

#### Grain size, moisture content, and pH of granulated bottom ash

Determining the physical properties of granulated bottom ash is essential due to its influence on the leaching of heavy metals and its environmental effects after landfilling or usage in municipal construction^46^. Therefore, the physical properties of bottom ash from waste incineration were investigated, and the results are presented below.

#### Particle size distribution of bottom ash

**Figure 1:**
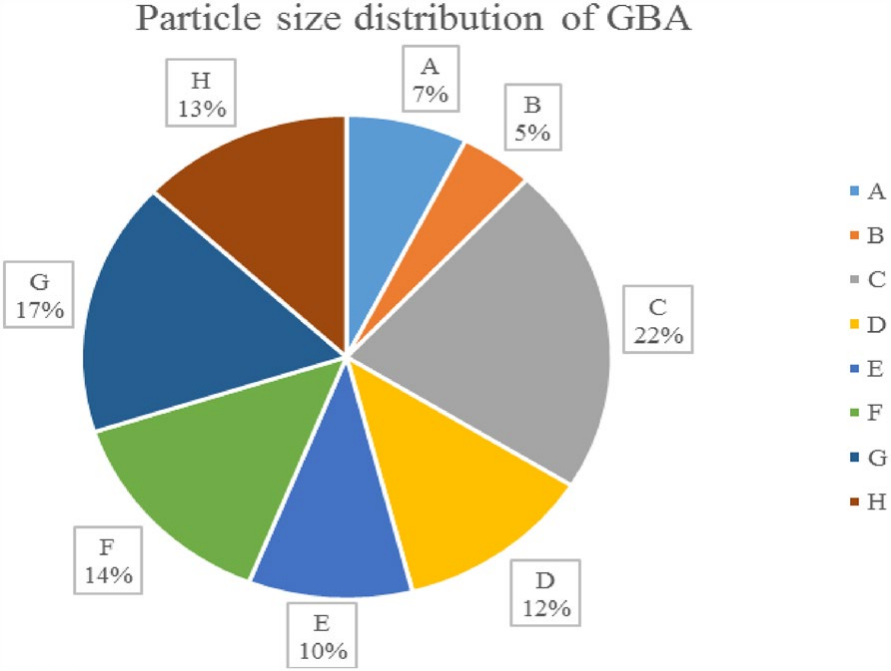
Particle size distribution of GBA.

Sample BA was granulated using steel sieves in the desired size ranges. Before sieving, coarse pieces such as broken glass, electrical wires and cables, ceramic pieces, and construction debris were manually separated from sample BA. As shown in Fig. 1, the obtained results showed that 7.4% of BA belonged to Group A, 4.5% to Group B, 22% to Group C, 12.1% to Group D, 10% to Group E, 14% to Group F, 17% to group G, and 13% to group H. Based on Supplementary Fig. S1 online, more than 70% of BA had a size smaller than 4 mm, consistent with studies conducted^27^. Previous studies show that particles smaller than 4 mm are suitable for road construction. Therefore, the particle size distribution of BA was in the range of 0–25 mm, similar to the particle size distribution of sand that falls into the preferable category and can be used in municipal constructions^25,47^.

Moreover, studies have shown that suitable particle sizes of BA for use as road construction materials are 0–2.36, 2.36–4.75, 0–4.75, and 0–9.5 mm nominal^26^. It has been reported that BA primarily consists of sand and gravel (60–90%), followed by silt and clay at 5–15%, and the remaining particles are more significant than 10 mm^11^. Particle size distribution is one of the critical parameters for selecting road construction materials. The particle size distribution of BA will vary depending on the location, time, and source of MSW^26^. Particle size distribution is one of the physical parameters of ash that increases heavy metal concentrations as particle size decreases due to the increased surface area. Therefore, the particle size distribution of ash is of particular importance^47^. Particle size distribution can be attributed to the air-to-MSW ratio and the degree of mixing in the waste incineration furnace^48^.

**Figure 2:**
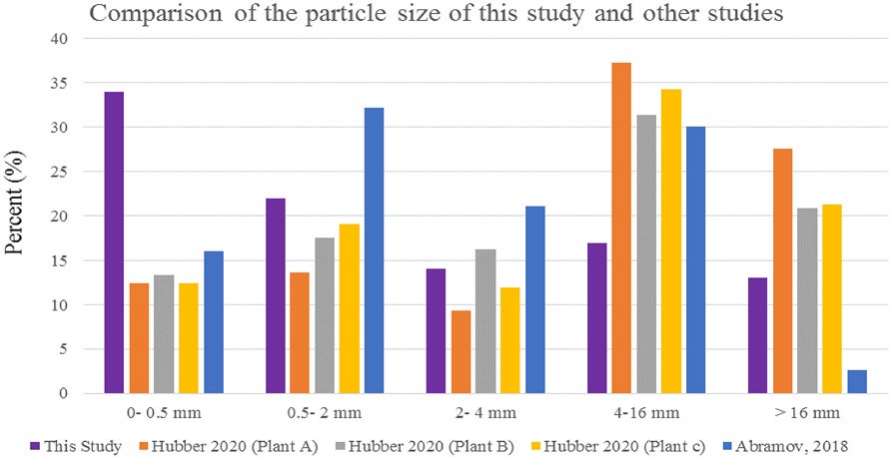
Comparison of the particle size of this study and other studies^15,49^.

As can be seen in Fig. 2, the distribution of particle size in this study was compared with other studies, and the difference in the percentage of particle distribution in each group is probably due to the difference in the composition of waste entering the waste incinerator and its management and operation conditions^15,49^.

#### Moisture content in size-granulated bottom ash

The moisture content of GBA was determined by placing it at a temperature of 105 °C until the weight stabilized, and the results are presented in Supplementary Fig. S2 online. As observed, the maximum moisture content was 2.27% in BA with particles smaller than 74 microns, and the moisture content decreased with the increasing particle size of the bottom ash. Some studies have examined the moisture content in FA^50–54^, while others have investigated the moisture content in Air Pollution Control Residual^55–57^, reporting values ranging from 0.27 to 3.5% by weight. The moisture content of waste incineration ash depends on the industrial equipment responsible for burning the waste, such as the combustion chamber, the presence of semi-dry scrubbers, and bag filters^56^. Given that moisture-loving substances like calcium salts are present in waste incineration residues, the moisture content of these residues will vary according to the amount of these substances^56^.

#### pH levels in granulated bottom ash particles

The pH of the GBA ash water was measured at one hour, one day, and seven days, and the changes were analyzed. Supplementary Fig. S3 online shows that the pH of the ash water in BA increased from 8.5 to around 12 within the first hour. There was a very slight increase in the pH of the ash water by the seventh day, which was hardly noticeable. Given that the pH of the deionized water used was 8.5, the changes in pH were due to the chemical compounds in the BA. The rapid increase in pH in the early hours is mainly due to the dissolution of Quicklime and Portlandite^41^.

On the other hand, although the composition of the Ash can varies greatly, high alkalinity in the BA (pH 10.5–13.5) was observed, which can be attributed to the presence of silica and sodium compounds^33,58^. The source of silica could be soda-lime glasses, the most common type of glass in the input waste and mainly composed of SiO_2_^58^. They are the primary cause of alkalinity in the BA.

### Chemical properties of size-granulated bottom ash

Determining metal, and heavy metals content, elemental characteristics, and mineralogical properties in size-granulated bottom ash.

#### Metals and heavy metals in size-granulated bottom ash

Since bottom ash generated from waste incineration is inherently very heterogeneous, the acid digestion method and ICP-OES and ICP-MASS devices were employed to determine the major and trace metals present^46,59^. Metal recovery is economically significant considering the particle size and metal content in Bottom Ash (BA)^27^. As shown in Table 1, the metals found in the waste incineration BA were evaluated in the size-classified groups GBA/A to GBA/H, categorized as elements with high concentrations, environmentally concerning elements, and precious elements.

**Table 1:** Classification of metals found in waste incineration BA.

Category	Elements
Significant elements (> 1000 mg/kg)	Ca, Fe, Al, Na, Mg, K, P, S, Ti, Mn
Elements of concern for the environment (2–1000 mg/kg)	As, Cu, B, Ba, Cr, Ga, La, Mo, Ni, Nb, Pb, Sn, Sr, Sb, Te, V, W, U, Y, Zn, Zr
Scarce elements in the environment (< 2 mg/kg)	Be, Bi, Ce, Co, Cs, Dy, Eu, Er, Gd, Ge, Hf, Ho, Hg, In, Ir, La, Lu, Li, Nd, Sm, Sc, Se, Ta, Tb, Th, Tl, Tm, Yb
Precious metals	Au, Ag

**Figure 3:**
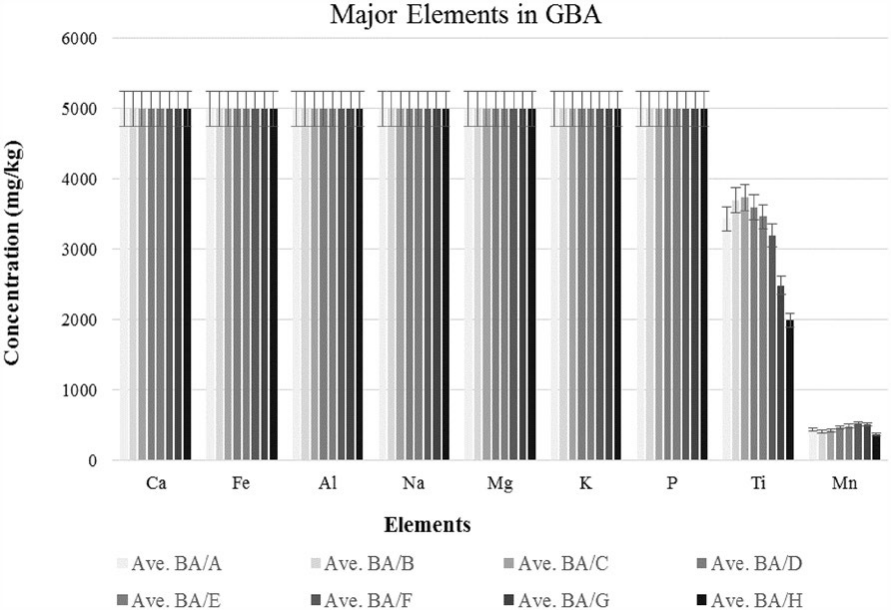
Major elements in particles.

Based on the results obtained from this study, which can be seen in Fig. 3 and Supplementary Table S2 online, and other studies, it was determined that the elements Ca, Fe, Na, Al, Mg, K, and P had the highest concentrations (> 5000 mg/kg) in BA and are considered significant elements^33^. Furthermore, it was observed that Al had the highest abundance (60%) in particles with a size of 6–20 mm^28^, followed by Ti with a variable concentration of 2000–3800 mg/kg in GBA/C particles, and Mn, which is present in almost all particle size ranges with a concentration of 300–500 mg/kg, are among the other main elements in BA.

**Figure 4:**
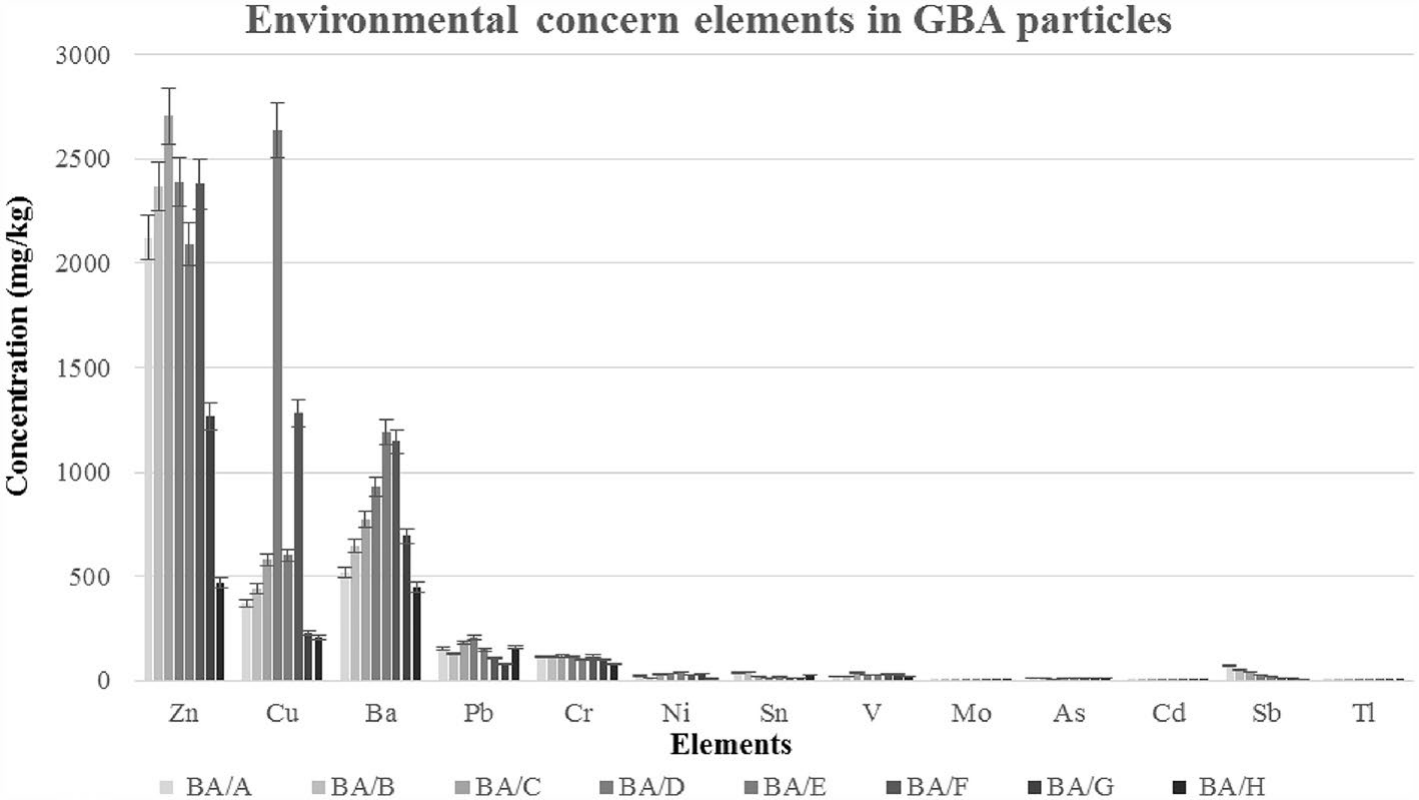
Environmental concern elements in GBA particles.

**Figure 5:**
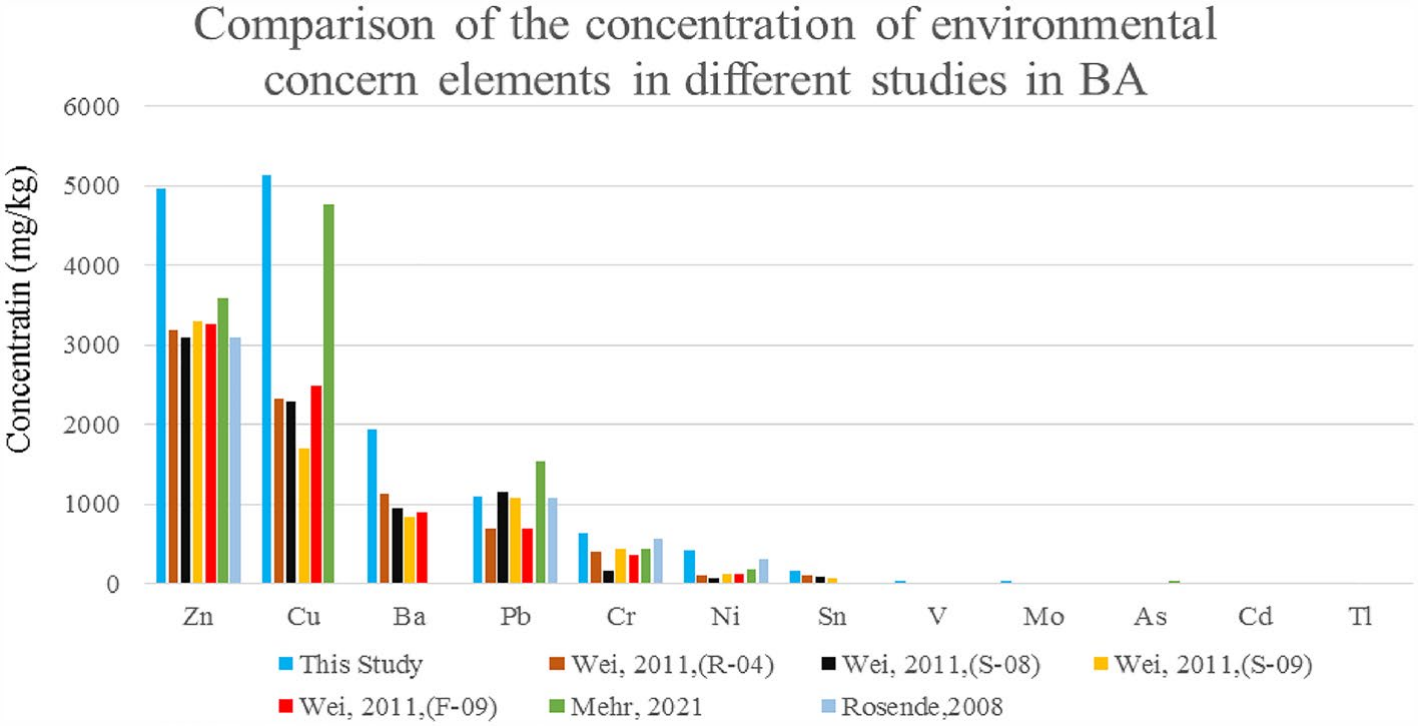
Comparison of the concentration of environmental concern elements in different studies^61–63^.

It was observed that the concentration of environmental concern elements significantly increased as the particle size of BA decreased, which can be attributed to changes in the mineralogy of the particles. As shown in Fig. 4 and Supplementary Table S3 online, Cu had a concentration of 200–2600 mg/kg in the GBA/D&F group, and it generally had a high concentration in particles smaller than 4 mm. Meanwhile, Zn had the highest concentration of 450–2700 mg/kg in the GBA/C group. Based on the results of this study and other studies, it was determined that the concentration of Zn and Cu increases significantly in particles smaller than 4 mm^33^. Ba had a concentration range of 400–1200 mg/kg, which was highest in the GBA/E group, while Pb had a concentration range of 120–200 mg/kg, which was almost uniformly distributed across all particle size ranges, except for the GBA/G group, which had the lowest concentration. The concentration of elements Cr, Ni, Sn, V, As, and Sb decreased with a gentle slope as the particle size increased, and the concentration of elements Mo, Cd, and Tl was detected to be less than 1 mg/kg. Overall, the concentration of elements Cr, Pb, Ni, and Sb increased as the particle size decreased^33^. By comparing the concentration of environmental concern elements in BA such as Zn, Cu, Cr, Ni, and Mo in this study and other studies, as shown in Fig. 5, it was observed that the concentration of these metals in this study is higher than in other studies. The need for complete recycling of the waste components entering the waste incinerator could be the reason for this. Also, the melting point of these metals is higher than the average temperature of burning MSW in the incinerator (about 850-950^ºC^), and the metal compounds entering the incinerator maintain their original form^27^. On the other hand, because the melting temperature of Zn is lower than the temperature applied in the incinerator to burn MSW, this metal can remain in the form of brass^27,60^.

The average concentration of heavy metals examined in BA particles smaller than 4 mm was compared to ecologically permissible levels for urban and garden uses, parks, open areas, and playgrounds, as shown in Table 2 and Supplementary Fig. S4 online. The results showed that the concentration of Sb in BA was higher than urban use and ecologically permissible levels, the concentration of Ba was higher than human and environmental health limits, ecologically permissible levels, and suitable soil levels, and the concentration of Cr was higher than ecologically permissible levels and suitable soil levels. Overall, the concentration of metals in BA was higher than the appropriate soil levels, indicating a high amount of rubber and plastic in the input waste, which needs to be treated before use in urban construction^64^.

**Table 2:** Heavy metal concentration in this study and comparison with standards.

Metals/metalloids	Permissible health levels mg/kg^65–67^			USEPA mg/kg^67,68^			Bottom ash (ave. concentration) (this study)
	Permissible ecological levels	Urban and garden use	Parks, open areas, and play gardens	Suitable soil limit	Limits for human health and environment	The extent that is necessary to improve the condition of the soil	
Antimony	20	30	–	–	–	–	37
Arsenic	20	100	200	20	30	50	11
Barium	400	5370	–	200	400	2000	868
Cadmium	3	20	40	1	5	20	0.5
Chromium	50	210	–	100	250	800	112
Copper	60	1000	2000	50	100	500	985
Lead	300	300	600	50	150	600	155
Manganese	500	1500	3000	–	–	–	462
Molybdenum	40	390	–	10	40	200	0.5
Nickel	60	600	600	50	100	500	27
Tin	50	46,900	–	20	50	300	26
Zinc	200	7000	14,000	200	500	3000	2343

**Figure 6:**
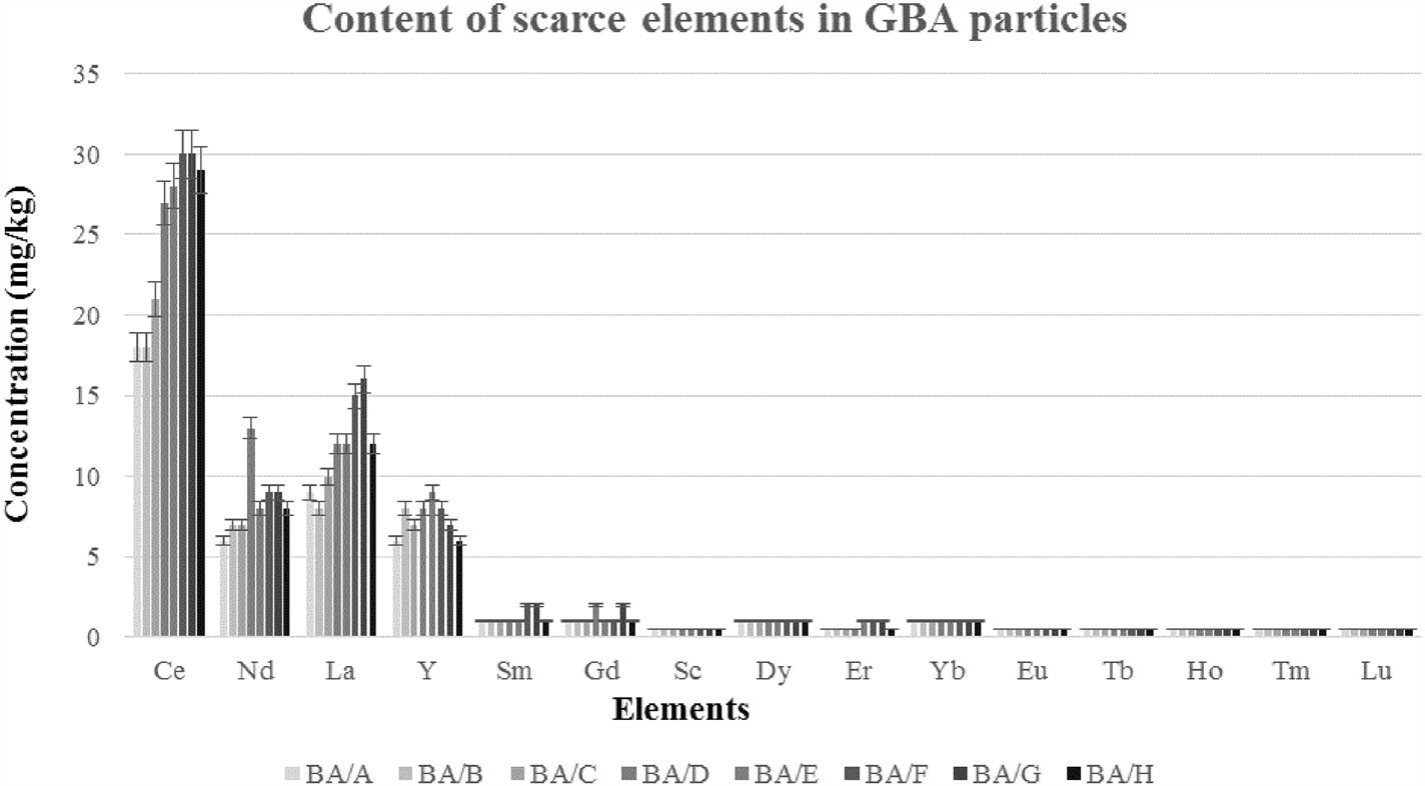
Content of scarce elements in GBA particles.

**Figure 7:**
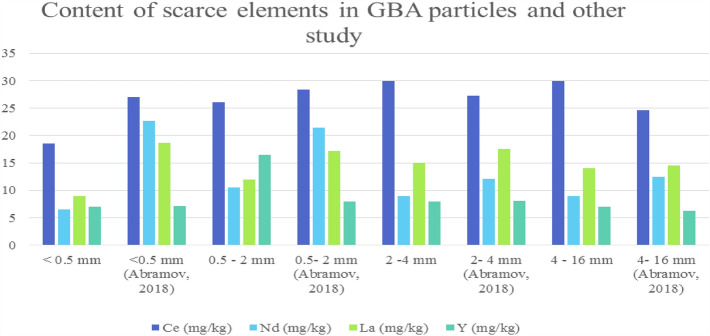
Comparison of scarce metals in GBA and other study^15^.

As per Fig. 6 and Supplementary Table S4 online, the scarce elements Ce in GBA/F&G group particles had the highest concentration at 30 mg/kg, Nd in GBA/D group with 13 mg/kg, La in GBA/F group with 15 mg/kg, and Y in GBA/E group particles with 9 mg/kg. Elements Sc, Eu, Tb, Ho, Hg, Tm, and Lu had the lowest concentration in BA, with less than 1 mg/kg, and in general, rare elements Ce, Nd, La, and Y have a higher concentration in the particles of 0.5–16 mm. As can be seen in Fig. 7, by comparing the rare metals Ce, Nd, La, and Y with Abramove's study^15^, it was observed that the concentration of these metals in different groups of GBA is similar to the results obtained in the mentioned study and almost the same.

**Figure 8:**
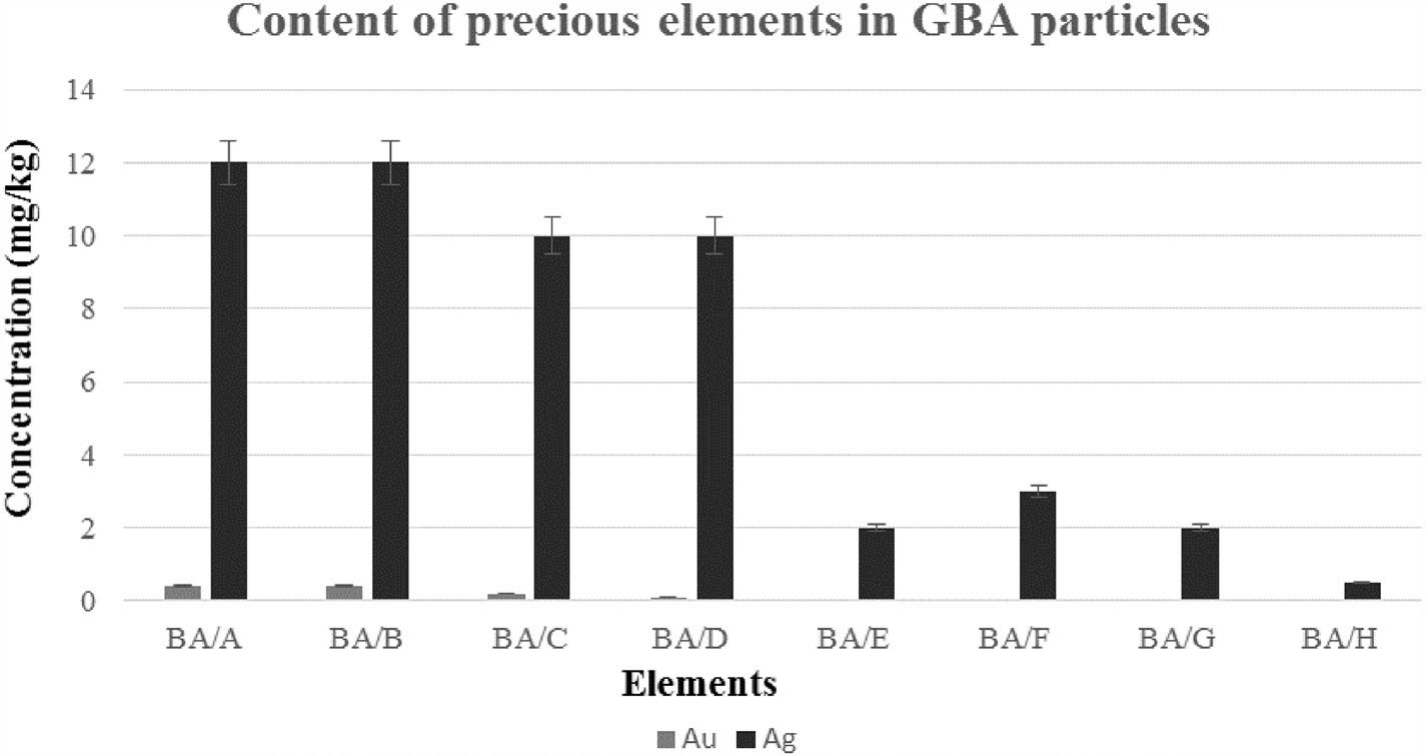
Content of precious elements in GBA particles.

Moreover, as shown in Fig. 8 and Supplementary Table S5 online, it was observed that the concentration of Au and Ag in GBA/A, and GBA/B, i.e., BA particles smaller than 0.125 mm, were 0.4 mg/kg and 12 mg/kg, respectively. With the increase in BA particle size, the concentration of these precious metals decreased, consistent with other studies that indicate these metals exist in particles smaller than 2 mm^30–32^. The source of precious metals in electrical and electronic equipment and the presence of gold and silver pieces in the input waste to the incinerator could be the reason for this^30^. The results of studies in this area have shown that fine particles are the most critical particles for recycling valuable metals and essential elements and removing harmful compounds. On the other hand, particles smaller than 4 mm constitute approximately half of the BA weight and are the most polluting part of BA. Therefore, these points should be considered in Municipal Construction^21^.

Supplementary Fig. S5 online shows that different metals had different concentrations in each GBA group. The elements Ca, Fe, Al, Na, Mg, K, and P had a high concentration and were the main elements in GBA particles, which was also confirmed by other studies^21,27^. The concentration of metals Zn, Ti, Cr, Sb, Ag, and Au decreased with increasing particle size, but the concentration of other elements was not related to particle size.

Based on the physicochemical properties of heavy metals, these elements are divided into several groups in the ash resulting from waste incineration. A. Hard-to-vaporize elements, such as Co, Cr, Cu, Mn, and Ni, mainly (about 90%) remain in the Bottom Ash during the waste incineration process. B. Elements that vaporize easily, including As, Pb, Zn, and Sn, of which 40–50% enter the fly ash, and the rest remain in the bottom ash. C. The element Cd is found in the bottom ash, fly ash, and flue gas stream at ratios of 10%, 85%, and 5%, respectively. D. The element in this group is Hg, which is highly volatile. Approximately 70% of Hg enters the gas stream, 5% is trapped in the bottom ash, and 25% enters the fly ash^68–70^. This classification is a good indicator of the behavior of heavy metals during the incineration process and gas stream cleaning^64^. Although the heavy metal content of fly ash is higher than that of bottom ash, more than 80% of As, Cr, Cu, and Ni metals, 74–94% of Zn, and 46–79% of Pb may remain in the bottom ash, which is primarily the waste incineration residue^64,71^. However, due to their high volatility, Cd and Hg enter fly ash at 47–73% and 60–100%, respectively^71^. Waste incinerators are considered an anthropogenic source of Hg emissions due to their high volatility, and the low efficiency of air pollution control devices in removing them from the generated vapors^64^. Additionally, due to food waste and wood residues, Na and K are observed at high concentrations in waste incinerator ash^2^. The pH level is another influential factor in the presence and concentration of metals in BA leachate. The solubility of metals depends on pH, but their solubility is different in different metals. Also, the solubility of trace elements is strongly dependent on pH^11^. The rapid dissolution of compounds such as quicklime (CaO) and Portlandite (Ca(OH)_2_) increases the pH to above 12.5. At this pH, the concentration of OH^-^ is significantly increased, which leads to the reaction with cations such as Zn^2+^ and Pb^2+^ and the production of metal hydroxides^27^. Also, studies showed that the concentration of trace elements such as Cu, Pb, Zn, and Cr is highly dependent on pH, and studies showed that at a pH higher than 12, the concentration of these metals increases strongly^72–76^, which is consistent with the results of this study. As the results obtained from the pH analysis in this study showed, a high pH above 12 leads to the dissolution of heavy metals and trace elements and their entry into the leachate resulting from washing BA and increasing the concentration of metals.

### Elemental characteristics (XRF) of size-granulated bottom ash

**Figure 9:**
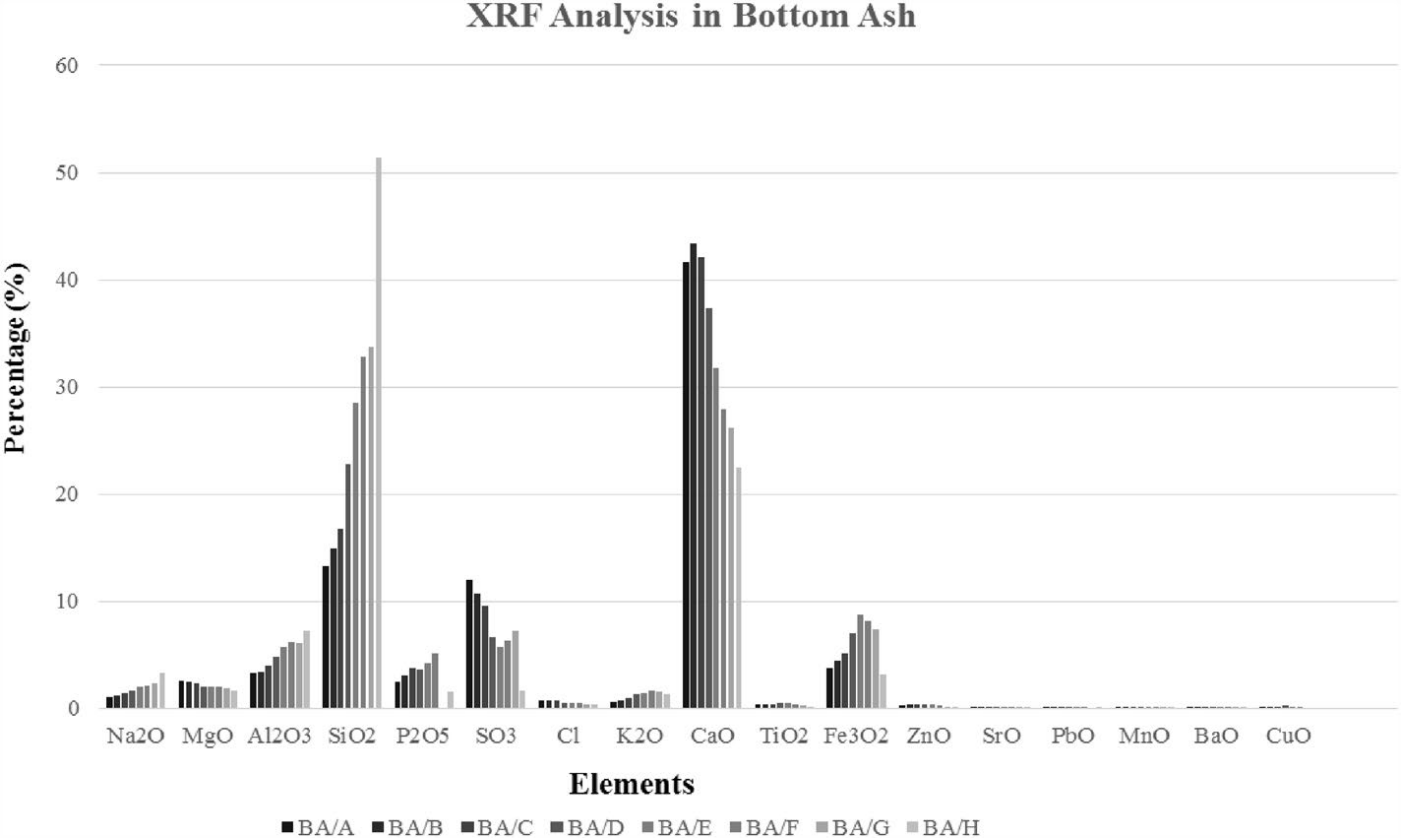
Mineral compositions and XRF analysis in GBA.

The chemical compositions and main elements in GBA (across eight different particle size ranges, GBA/A to GBA/H) were determined using X-Ray Fluorescence (XRF). The results are shown in Fig. 9 and Supplementary Table S6 online. Based on the findings of this study and other research, it was determined that chemical compositions have a direct relationship with particle size. As the bottom ash particle size increases, silica and sodium compounds increase^33,58^, while calcareous compounds decrease. The presence of glass in the waste input to the waste incinerator could be the reason for this. Soda-lime glasses, the most common type of glass found in waste, are mainly composed of SiO_2_ and lesser amounts of Na_2_O and CaO, with deficient levels of MgO, K_2_O, Al_2_O_3_, and TiO_2_. Thus, these chemical compositions in the bottom ash are related to the presence of glass in the waste^58^.

Iron oxides are mainly observed in medium and coarse particles (16–0.5 mm). Due to the possibility of manual and magnetic separation of iron metals in the recycling plant and the entry of small amounts of them into the waste incinerator, as well as their high melting temperature and hardness, these compounds are not observed in smaller particles. Batteries in the waste are the main cause of heavy metals, such as lead and zinc, in the ash. Zn is observed in small to medium particles, while Pb is in larger particles. The difference in the boiling points of these two substances could be the reason for this. Although the melting points of Zn and Pb are lower than the incineration temperature in the waste incinerator, the boiling point of lead is much higher, and zinc is close to the incineration temperature (Pb: 1740^ºC^ and Zn: 907^ºC^).

For this reason, after cooling, Pb forms larger particles, and Zn forms smaller particles^58,68^. On the other hand, elements like Cu, Zn, and Pb can enter the gaseous phase during combustion, forming fine particles with a high metal concentration. These are also observed in Fly Ash^77^.

Another compound observed in bottom ash is titanium, which is present in fine bottom ash particles due to its use in the paint and cosmetic industries; however, as the particle size of bottom ash increases, chlorine, and sulfur decrease. Studies have shown that changing the particle size does not change the weight percentage of aluminum oxide^58^. However, the present study observed that an increase in particle size led to an increase in aluminum oxide. This could be because of the temperature at which the waste is incinerated in waste-to-energy facilities.

During the incineration process, liquid aluminum is formed, which may absorb non-combustible particles and form complex compounds within a range of different particle sizes^58^.

**Figure 10:**
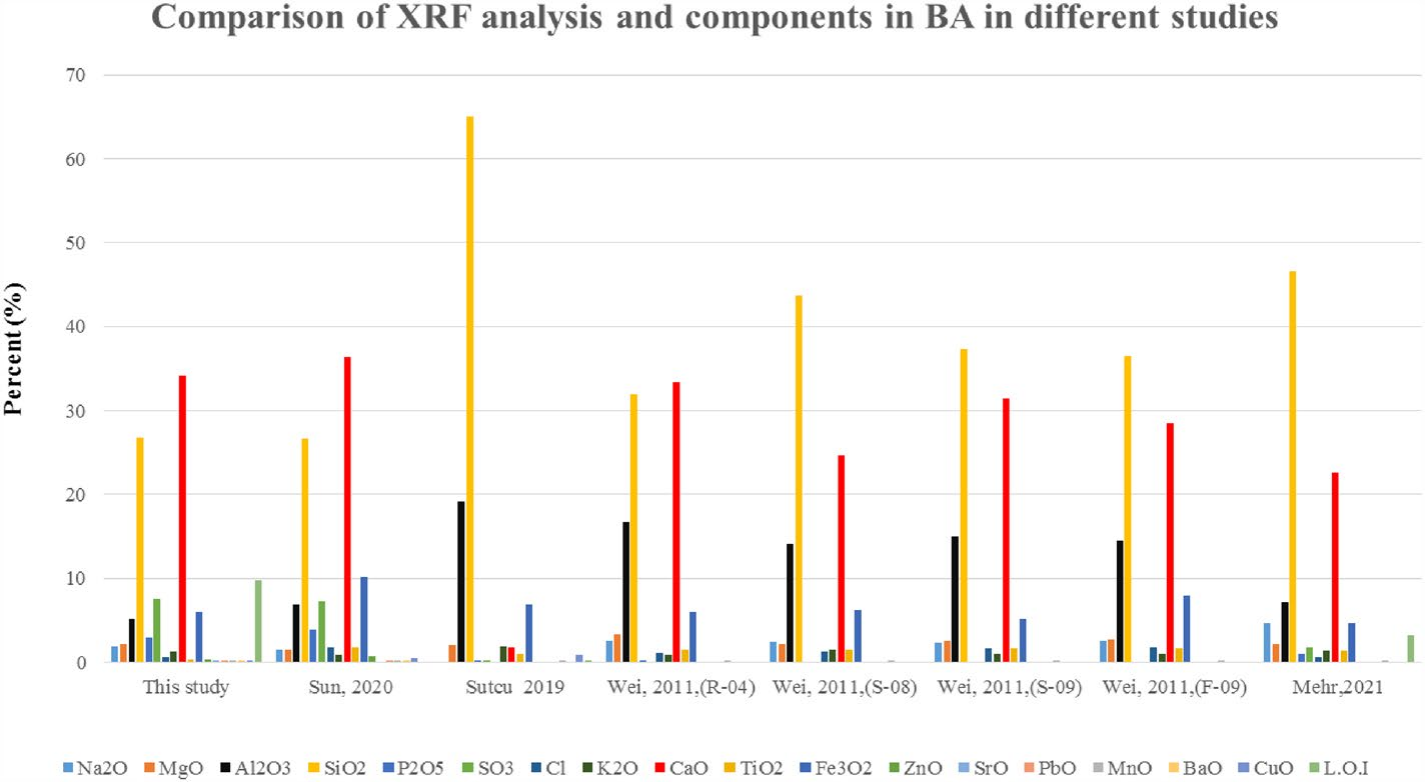
Comparison of XRF analysis and components in BA in different studies^41,62,63,78^.

The overall comparison of the elements in BA in this study and other studies, which can be seen in Fig. 10, showed that in this study, SiO_2_ has a lower concentration than other studies, and the concentration of other oxides is similar to other studies^41,62,63^. The composition of incoming waste, waste incinerator design, and seasonal changes cause changes in the concentration of elements and oxides^62^.

### Loss on ignition analysis of size-granulated bottom ash content

**Figure 11:**
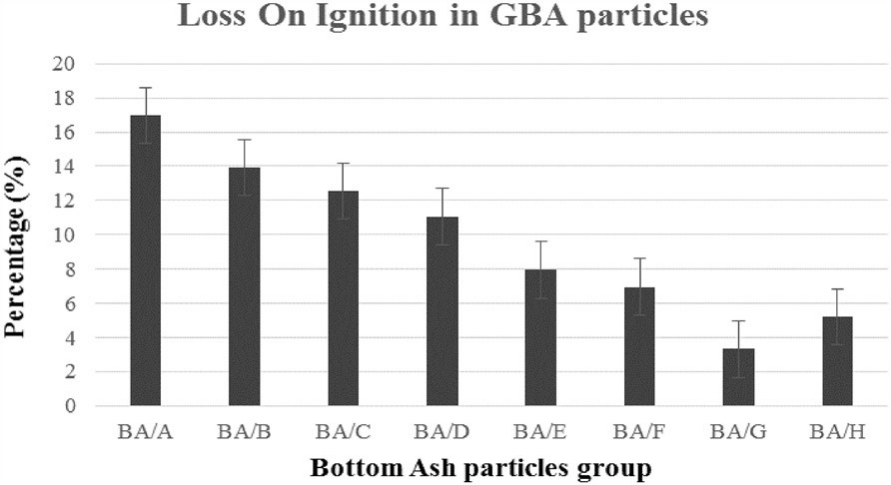
Loss on the ignition I GBA particles.

The LOI (loss on ignition) index, which represents the reduction due to combustion, indicates the amount of organic matter in the ash. As shown in Fig. 11, an increase in the particle size of bottom ash led to a decrease in the LOI value. The data/observation indicates that the amount of organic matter decreased as the particle size increased^79^. According to the French Ministry of the Environment regulations, if the LOI value is less than 5%, the bottom ash is classified as non-hazardous (Category V) and can be used in Municipal construction^79^. However, the LOI value in the bottom ash studied, with a particle size of 0.075–4 mm, was higher than 5%, which cannot be considered non-hazardous. Nevertheless, particles in Group GBA/G, with a particle size of 4–16 mm and an LOI value of approximately less than 5%, are considered non-hazardous and can be used in Municipal construction. The LOI value in particles of Group A was high and similar to other studies^80^.

### Mineralogical characteristics (XRD) of size-granulated bottom ash

The XRD analysis results for bottom ash in eight groups (GBA/A to GBA/H), and the presence of the mineral compounds are shown in Table 3. As observed, the minerals phases Calcite (CaCO_3_), Quartz (SiO_2_), Gehlenite (Ca_2_Al_2_SiO_7_), Larnite (Ca_2_SiO_4_), Ettringite (Ca_6_Al_2_(SO_4_)_3_(OH)_12_.26H_2_O), Magnetite (Fe_2_O_3_) and Anidrit (CaSO_4_) are the main components in all GBA groups; they can be identified in any grain size fraction^15,41^. For better examination, one of the groups is introduced as a representative index, and its results are compared with other studies. Therefore, Group G, with a 4–16 mm particle size, is introduced as the index group^26^. In this group, Calcite, Gehlenite, Larnite, and Quartz minerals, have the highest amounts among other groups. However, the main structure of this group is Calcite and Quartz, which is consistent with the results of other studies^26,34,80,81^.

**Table 3:** XRD analysis and crystalline phases in GBA.

Crystalline phase	BA/A	BA/B	BA/C	BA/D	BA/E	BA/F	BA/G	BA/H
Calcite (CaCO_3_)	✓	✓	✓	✓	✓		✓	✓
Portlandite (Ca(OH)_2_)	✓	✓	✓	✓	✓	✓	✓	
Calcium sulfate (CaSO_4_)	✓	✓	✓	✓	✓	✓	✓	
Ettringite (Ca_6_Al_2_(SO_4_)_3_(OH)_12_⋅26H_2_O)	✓	✓	✓	✓	✓			
Calcium Silicate (Ca_3_SiO_5_)	✓	✓	✓	✓	✓	✓	✓	
larinite (Ca2SiO4)	✓	✓	✓	✓	✓	✓	✓	✓
Gehlenite (Ca_2_Al_2_SiO_7_)	✓	✓	✓	✓	✓	✓	✓	✓
Quartz (SiO_2_)	✓	✓	✓	✓	✓	✓	✓	✓
Hydroxylapatite (Ca_5_(PO_4_)_3_(OH))	✓	✓	✓	✓	✓	✓	✓	
Dolomite (CaMg(CO_3_)_2_/CaO⋅MgO⋅2CO_2_)			✓					
Albite.calcian.ordered ((Na,Ca)Al(Si,Al)_3_O_8_)			✓	✓	✓	✓	✓	✓
Microcline, intermediate (KAlSi_3_O_8_)				✓				✓
Cristobalite (SiO_2_)						✓		
Magnetite (Fe_3_O_4_)						✓	✓	
Augite, aluminian (Ca(Mg,Fe^3+^,Al)(Si,Al)_2_O_6_)						✓	✓	
Magnetite (Fe_3_O_4_)							✓	

Furthermore, Portlandite (Ca(OH)_2_) and Hydroxylapatite (Ca_5_(PO_4_)_3_(OH)) are among the main components of GBA/G. In addition to these minerals, Anhydrite (CaSO_4_), Calcium Silicate (Ca_3_SiO_5_), and Magnetite (Fe_3_O_4_) can also be seen in the structure of this group of particles. With the increase in calcium content, other calcium-containing compounds such as Anhydride (CaSO_4_) decreased, consistent with the results obtained by other scientists^25,82,83^, and shows that the mineralogical composition of different countries is almost equal^25^. During the quenching process, Portlandite was formed when CaO reacted with water. Ettringite may also be produced due to the alkaline environment created by CaO and the presence of SO_4_^2^ and Al^3+^ in BA after the quenching process^41^. The results of this study are consistent with other studies^41,58^.

In some cases, the formation of (hydro) oxides in the burning process is attributed to the alkalinity of the solid residue^15^. Based on the conducted studies, it can be observed that the main minerals of BA are Calcite and Quartz, which remain unchanged in different locations, times, and particle size variations of BA^26^. Moreover, the presence of SiO_2_ in BA makes its structure similar to the structure of mineral compounds used in road construction^26,79,84^. Therefore, BA can be used for road construction, and the results also show that the minerals present in the BA structure are unrelated to the particle size^26^. The graphs related to the XRD analysis in GBA can be seen in Supplementary Fig. S6 online. As observed in the graphs related to all eight GBA groups, the mineral phases in the samples cannot be separated well due to their overlap, and most of the minerals are common in all groups. However, a few of them in some groups have higher concentrations.

Another effective parameter in BA composition is pH. As observed, Quartz, Calcite, Portlandite, and Ettringite are the main components of BA. Calcite raises the pH above 8, and Ettringite increases the pH to above 10, and the main reason for the high pH in BA is Quicklime and Portlandite compounds, which increase the pH to above 12^85^. Due to the rapid dissolution of Quicklime and Portlandite, these two compounds increase the pH to above 12.5 in the early hours, and the increase in pH causes the dissolution of other metals and their release into the environment^41^.

In summary, the XRD analysis of granulated bottom ash indicates that Calcite and Quartz are the primary minerals in all groups, with varying percentages in different groups. The results suggest that BA can be used for road construction, and the minerals in the BA structure are unrelated to the particle size. Overall, the study provides insights into the mineralogical characteristics of granulated bottom ash, which could benefit its sustainable management and utilization.

## Conclusion

Using incinerators for waste management and energy recovery from Municipal Solid Waste (MSW) produces other by-products, such as bottom ash and fly ash. These by-products require proper management due to their inherent characteristics and negative environmental impacts. Incineration by-products can be used in various municipal and civil construction and road-building applications, but their use requires careful examination of their properties and appropriate treatment. Due to heavy metals, toxic elements, and salts harmful to humans and the environment, Bottom Ash (BA) needs proper treatment before use or landfilling. In Iran, landfilling is the routine method for disposing of municipal waste, and other technologies, especially incineration, are less frequently used. However, in recent years, due to the focus on energy recovery from municipal waste and other benefits of using incinerators, this technology has been expanding and being utilized in some cities, including Tehran, alongside other waste management technologies such as recycling, compost production, and landfilling. To manage and use them properly, it was necessary to thoroughly investigate the physicochemical properties of the residues from Tehran's municipal waste incinerators. One important parameter to investigate is the particle size of BA. Results showed that approximately 70% of the particles are smaller than 4 mm, making them suitable for civil construction and road-building applications. In general, the distribution of BA particle sizes ranged from 0 to 25 mm, similar to the distribution of sand particle sizes, and can be used in municipal construction. The pH investigation revealed that BA has high alkalinity (pH = 10.5–13.5) due to silicate and sodium compounds from soda-lime glass in the input waste. The resulting leachate has a high pH, leading to the dissolution of heavy metals (lead and zinc) and their release into the environment. It also increases the acid-neutralizing capacity to reduce its alkalinity.

The ICP-MASS analysis of metal content showed that the elements Ca, Fe, Na, Al, Mg, K, and P have the highest concentrations (> 5000 mg/kg) in BA and are considered the primary elements. In contrast, the heavy metals Zn, Cu, Ba, Pb, Cr, Ni, Sn, V, Mo, As, Cd, and Tl have high concentrations (2–1000 mg/kg) and exhibit environmental toxicity. The concentration of heavy metals increases with the decrease in particle size, especially in finer particles of BA, where higher concentrations of lead and zinc are found. Rare earth elements Ce, Nd, La, Y, Sm, Gd, Sc, Dy, Er, Yb, Eu, Tb, Ho, Tm, and Lu were also identified in BA with concentrations < 2 mg/l. In finer particles (< 1 mm), gold (0.1–0.4 mg/kg) and silver (10–12 mg/kg) were found in notable concentrations.

XRF analysis of the crystalline phases showed that the main elements present in BA are silica, calcium, and sodium, which have higher concentrations in finer particles.

XRD analysis revealed that the minerals Calcite, Quartz, Gehlenite, and Larinite are the main components in all BA groups, but their concentrations vary among different particle groups. The results of this study showed that the concentration of heavy metals in BA is very high, and landfilling or using them in road construction and civil activities can cause environmental issues due to the potential leakage of heavy metals. Treating the BA before using or disposing of it in special landfills or monofills.

### Supplementary Information

Supplementary Information.

## Data Availability

All data generated or analysed during this study are included in this published article and its supplementary information files.
