# Supplementary material for: Analysis of heavy metal, rare, precious, and metallic element content in bottom ash from municipal solid waste incineration in Tehran based on particle size
Source: Sci Rep. 2023 Sep 25;13:16044. doi: 10.1038/s41598-023-43139-1 (PMC10520020; doi:10.1038/s41598-023-43139-1)
Supplement: Supplementary file 1 — Supplementary Information. [file 41598_2023_43139_MOESM1_ESM.doc]

**Analysis of Heavy Metal, Rare, Precious, and Metallic Element Content in Bottom Ash from Municipal Solid Waste Incineration in Tehran Waste Processing Complex, Iran, Based on Particle Size**

Amir Hossein Mahvi, Kamyar Yaghmaeian, Ramin Nabizadeh, Masoumeh Beikmohammadi

**Supplementary file**

Table S1 - Nominal parameters of Tehran's municipal waste incinerator

| Operating conditions:  Process type: Gasification  Capacity: 200 tons per day  Electric power generated: 3 megawatts of electricity  Air pollution control device: Fabric Filter  Solid waste management: Landfill disposal  Additives in the scrubber unit:  Ca (OH)_2_ slurry  Activated carbon  NOx control with NH_4_OH  Bottom ash  Residues from the air pollution control system |
| --- |


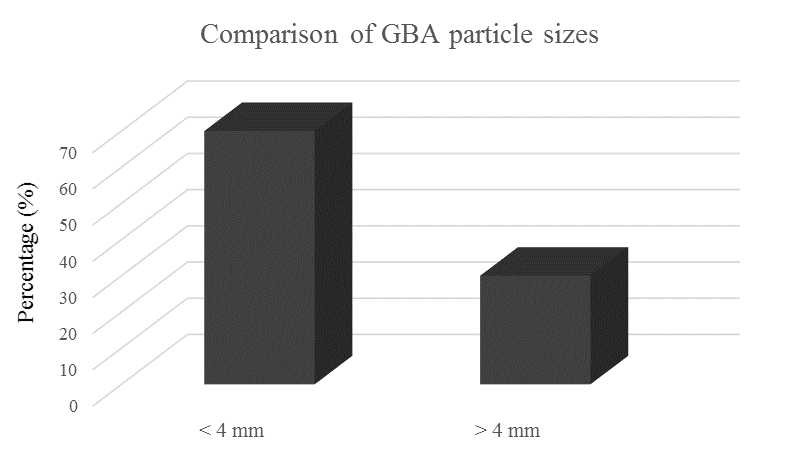


Figure S1: Comparison of GBA particle sizes


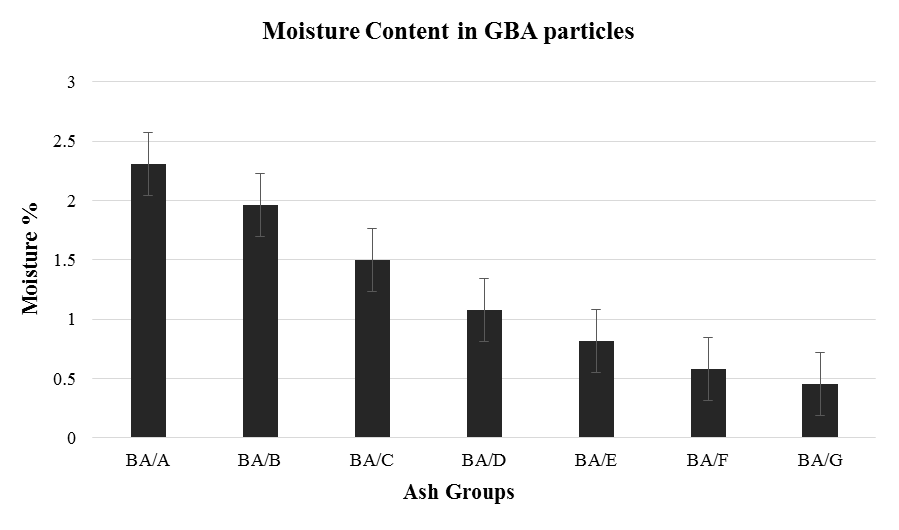


Figure S2: Moisture content in GBA


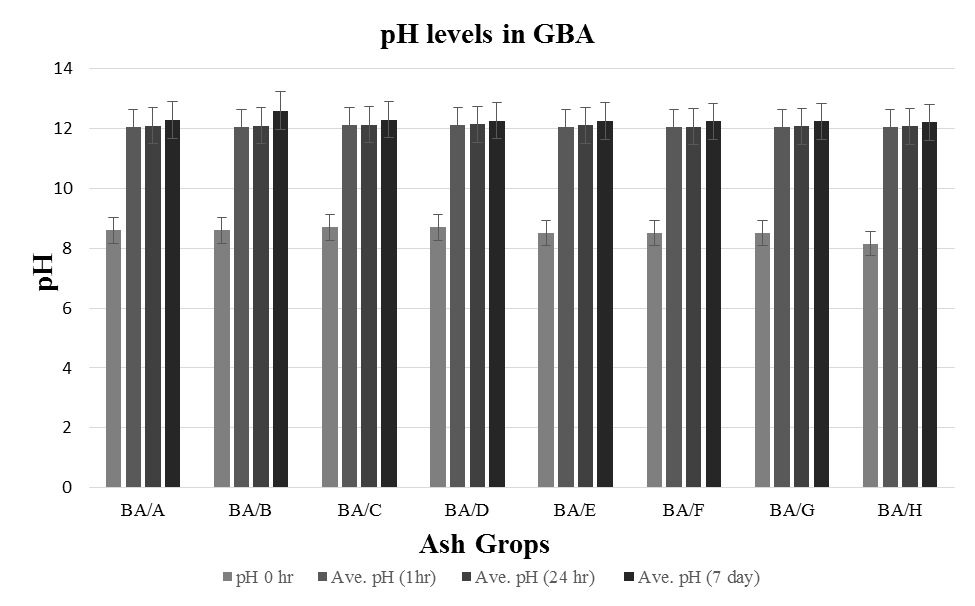


Figure S3: pH levels in GBA

Table S2: Major elements in GBA particles

| **Major elements (quantified by XRF), mg/kg** | | | | | | | |  |
| --- | --- | --- | --- | --- | --- | --- | --- | --- |
|  | BA/A | BA/B | BA/C | BA/D | BA/E | BA/F | BA/G | BA/H |
| **Ca** | 5000 | 5000 | 5000 | 5000 | 5000 | 5000 | 5000 | 5000 |
| **Fe** | 5000 | 5000 | 5000 | 5000 | 5000 | 5000 | 5000 | 5000 |
| **Al** | 5000 | 5000 | 5000 | 5000 | 5000 | 5000 | 5000 | 5000 |
| **Na** | 5000 | 5000 | 5000 | 5000 | 5000 | 5000 | 5000 | 5000 |
| **Mg** | 5000 | 5000 | 5000 | 5000 | 5000 | 5000 | 5000 | 5000 |
| **K** | 5000 | 5000 | 5000 | 5000 | 5000 | 5000 | 5000 | 5000 |
| **P** | 5000 | 5000 | 5000 | 5000 | 5000 | 5000 | 5000 | 5000 |
| **Ti** | 3432 | 3695 | 3767 | 3590 | 3463 | 3195 | 2488 | 1995 |
| **Mn** | 437 | 418 | 431 | 471 | 492 | 523 | 511 | 371 |

Table S3: Environmental concern elements in GBA particles

| **Elements of typical environmental concern (quantified by MP-AES), mg/kg** | | | | | | | | |
| --- | --- | --- | --- | --- | --- | --- | --- | --- |
|  | BA/A | BA/B | BA/C | BA/D | BA/E | BA/F | BA/G | BA/H |
| **Zn** | 2122 | 2367 | 2706 | 2389 | 2092 | 2380 | 1267 | 469 |
| **Cu** | 367 | 440 | 579 | 2639 | 600 | 1283 | 227 | 206 |
| **Ba** | 517 | 646 | 775 | 930 | 1192 | 1147 | 693 | 447 |
| **Pb** | 156 | 129 | 182 | 205 | 147 | 108 | 77 | 158 |
| **Cr** | 114 | 115 | 116 | 113 | 100 | 117 | 102 | 76 |
| **Ni** | 22 | 12 | 29 | 34 | 40 | 25 | 33 | 8 |
| **Sn** | 39 | 39 | 40 | 10 | 18 | 11 | 10 | 25 |
| **V** | 17 | 20 | 35 | 26 | 27 | 30 | 29 | 21 |
| **Mo** | 0.5 | 0.5 | 0.5 | 0.5 | 0.5 | 0.5 | 0.5 | 0.5 |
| **As** | 12 | 10 | 7 | 12 | 11 | 12 | 12 | 14 |
| **Cd** | 0.5 | 0.5 | 0.5 | 0.5 | 0.5 | 0.5 | 0.5 | 0.5 |
| **Sb** | 74 | 50 | 39 | 26 | 20 | 12 | 9 | 6 |
| **Tl** | 0.5 | 0.5 | 0.5 | 0.5 | 0.5 | 0.5 | 0.5 | 0.5 |


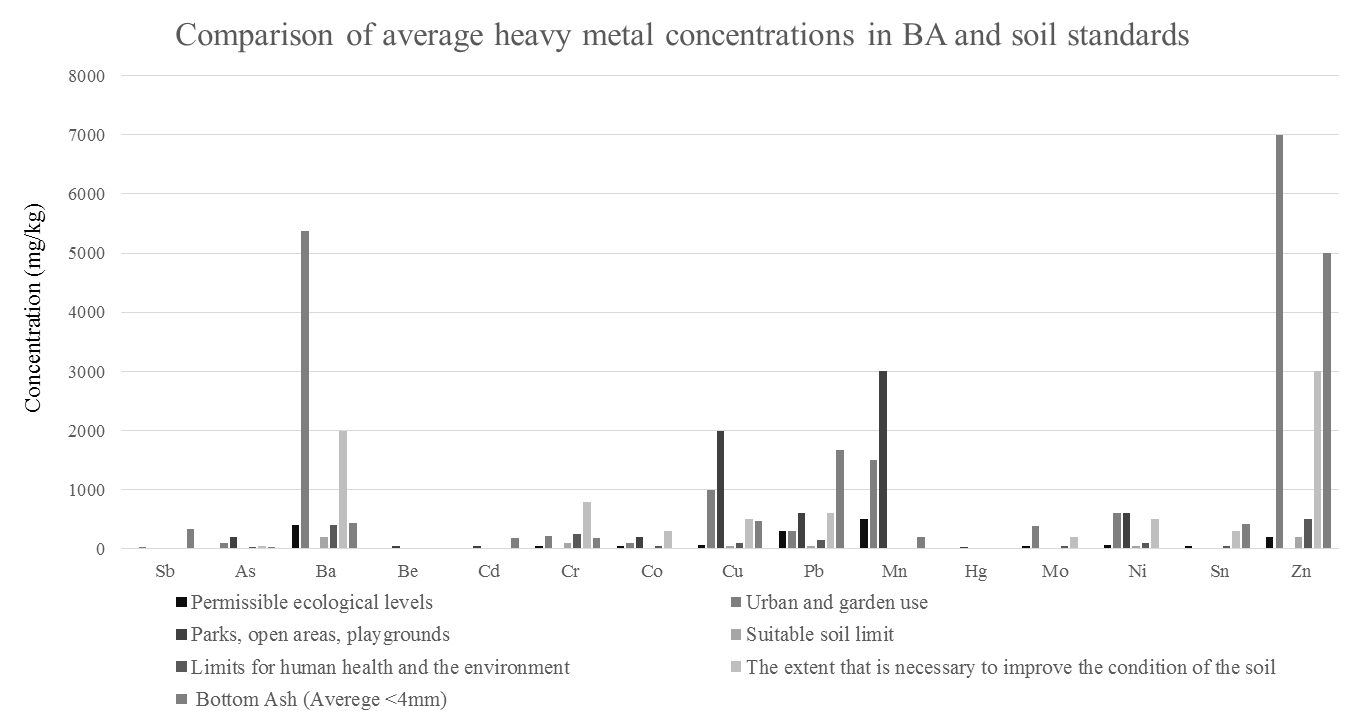


Figure S4 - Comparison of average heavy metal concentrations in BA and soil standards

Table S4: Content of scarce elements in GBA particles

| **Rare Earth Elements (REEs; quantified by ICP-QMS), mg/kg** | | | | | | | | |
| --- | --- | --- | --- | --- | --- | --- | --- | --- |
|  | BA/A | BA/B | BA/C | BA/D | BA/E | BA/F | BA/G | BA/H |
| **Ce** | 18 | 17 | 21 | 26 | 26 | 30 | 30 | 27 |
| **Nd** | 6 | 7 | 7 | 13 | 8 | 9 | 9 | 8 |
| **La** | 9 | 8 | 10 | 12 | 12 | 15 | 14 | 12 |
| **Y** | 6 | 7 | 7 | 8 | 9 | 8 | 7 | 6 |
| **Sm** | 1 | 1 | 1 | 1 | 1 | 2 | 2 | 1 |
| **Gd** | 1 | 1 | 1 | 2 | 1 | 1 | 2 | 1 |
| **Sc** | 0.5 | 0.5 | 0.5 | 0.5 | 0.5 | 0.5 | 0.5 | 0.5 |
| **Dy** | 1 | 1 | 1 | 1 | 1 | 1 | 1 | 1 |
| **Er** | 0.5 | 0.5 | 0.5 | 0.5 | 1 | 1 | 1 | 0.5 |
| **Yb** | 1 | 1 | 1 | 1 | 1 | 1 | 1 | 1 |
| **Eu** | 0.5 | 0.5 | 0.5 | 0.5 | 0.5 | 0.5 | 0.5 | 0.5 |
| **Tb** | 0.5 | 0.5 | 0.5 | 0.5 | 0.5 | 0.5 | 0.5 | 0.5 |
| **Ho** | 0.5 | 0.5 | 0.5 | 0.5 | 0.5 | 0.5 | 0.5 | 0.5 |
| **Tm** | 0.5 | 0.5 | 0.5 | 0.5 | 0.5 | 0.5 | 0.5 | 0.5 |
| **Lu** | 0.5 | 0.5 | 0.5 | 0.5 | 0.5 | 0.5 | 0.5 | 0.5 |

Table S5: Content of precious elements in GBA particles

| **Precious metals (quantified by ICP-OES), mg/kg** | | | | | | | | |
| --- | --- | --- | --- | --- | --- | --- | --- | --- |
|  | BA/A | BA/B | BA/C | BA/D | BA/E | BA/F | BA/G | BA/H |
| **Au** | 0.4 | 0.4 | 0.2 | 0.1 |  |  |  |  |
| **Ag** | 12 | 12 | 10 | 10 | 2 | 3 | 2 | 0.5 |


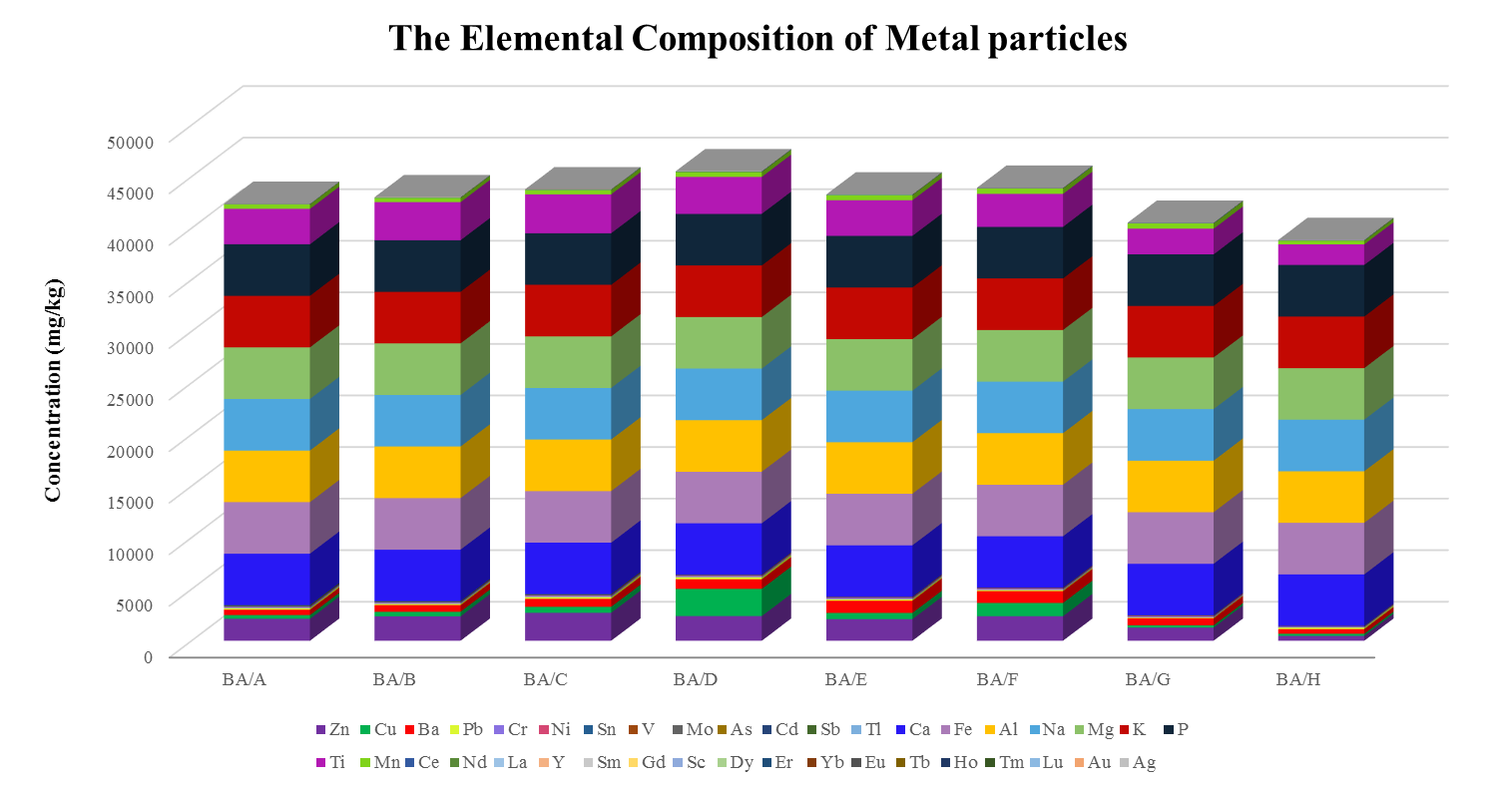


Figure S5- Comparison of metal content in GBA

Table S6: Mineral composition and XRF analysis in GBA

| **Oxide** | **BA/A** | **BA/B** | **BA/C** | **BA/D** | **BA/E** | **BA/F** | **BA/G** | **BA/H** |
| --- | --- | --- | --- | --- | --- | --- | --- | --- |
| **Na_2_O** | 1.1 | 1.2 | 1.4 | 1.7 | 2 | 2.1 | 2.3 | 3.3 |
| **MgO** | 2.6 | 2.5 | 2.3 | 2 | 2.05 | 2 | 1.9 | 1.7 |
| **Al_2_O_3_** | 3.3 | 3.4 | 3.95 | 4.78 | 5.7 | 6.17 | 6.1 | 7.2 |
| **SiO_2_** | 13.3 | 14.86 | 16.8 | 22.85 | 28.49 | 32.84 | 33.72 | 51.45 |
| **P_2_O_5_** | 2.5 | 3.1 | 3.7 | 3.6 | 4.2 | 5.2 | 0 | 1.5 |
| **SO_3_** | 11.96 | 10.7 | 9.58 | 6.7 | 5.7 | 6.3 | 7.2 | 1.7 |
| **Cl** | 0.69 | 0.72 | 0.68 | 0.52 | 0.47 | 0.44 | 0.4 | 0.33 |
| **K_2_O** | 0.63 | 0.71 | 0.93 | 1.3 | 1.4 | 1.6 | 1.5 | 1.3 |
| **CaO** | 41.6 | 43.4 | 42.1 | 37.3 | 31.7 | 27.9 | 26.2 | 22.5 |
| **TiO_2_** | 0.42 | 0.39 | 0.4 | 0.48 | 0.45 | 0.36 | 0.31 | 0.18 |
| **Fe_3_O_2_** | 3.7 | 4.5 | 5.2 | 7 | 8.7 | 8.2 | 7.3 | 3.2 |
| **ZnO** | 0.31 | 0.39 | 0.38 | 0.34 | 0.36 | 0.31 | 0.16 | 0.06 |
| **SrO** | 0.12 | 0.1 | 0.1 | 0.12 | 0.11 | 0.08 | 0.09 | 0.08 |
| **PbO** | 0.02 | 0.02 | 0.02 | 0.02 | 0.02 | 0.02 | 0 | 0.02 |
| **MnO** | 0.05 | 0.05 | 0.05 | 0.06 | 0.06 | 0.07 | 0.07 | 0.04 |
| **BaO** | 0.07 | 0.08 | 0.1 | 0.11 | 0.16 | 0.16 | 0.09 | 0.06 |
| **CuO** | 0.04 | 0.06 | 0.08 | 0.3 | 0.08 | 0.16 | 0 | 0 |

| A |
| --- |
| B |
| C |
| D |
| E |
| F |
| G |
| H |
| Figure S6- Graphs related to XRD analysis in GBA in particles of group A, B, C, D, E, F, G, H |
